# From the perspective of spatial justice and temporal justice a study on social stratification of physical activity participation among Chinese residents—empirical analysis based on CGSS2023

**DOI:** 10.3389/fpsyg.2026.1742881

**Published:** 2026-02-09

**Authors:** Wei Liu, XiaoPeng Shang, Yongtao Zhang

**Affiliations:** 1 Postdoctoral Mobile Station, Chengdu Sport University, Chengdu, China; 2 National Fitness Innovation and Collaboration Center, Anhui Professional Institute of Sport, Hefei, China; 3 Department of Public Health Emergency Response, Zhejiang Provincial Center for Disease Control and Prevention, Hangzhou, China

**Keywords:** cultural capital, health inequity, physical activity, social stratification, spatial justice, temporal justice

## Abstract

**Introduction:**

Physical activity is one of the important ways to promote public health and implement the “Healthy China” strategy. Currently, physical activity has become an evident difference in participation between various social classes; this is becoming a key part of inequality in health. The purpose of this study is to explore how social stratification affects Chinese residents’ participation in physical activities, so provide a theoretical basis for formulating more fair and effective public health policies.

**Methods:**

This study provides an analytical framework from the three dimensions of space, time and capital. The 2023 China General Social Survey (CGSS) data with a sample size of 2129 is taken for ordered logistic regression model to explore how social stratification affects residents’ participation in physical activities through various channels. This method systematically investigates how independent and interactive effects of various social factors affect physical activity frequency.

**Results:**

1) Significant spatial gradient effect: residents’ physical activity frequency shows a systematic increase from the countryside, towns, urban fringes to the center of the city. 2) Time constraints are everywhere: For every hour more you work, exercise becomes less across all regions; 3) Cultural capital makes a big difference: Controlling for income, education years show strong positive effect on physical activity even after that; 4) The “gym paradox” was found - people who regularly use gyms for exercise actually do the least amount of exercise.

**Discussion:**

This study shows the various levels of mechanisms through which social stratification affects participation in physical activities. Spatial differences are not only the “compositional effect” of socioeconomic factors but also an independent “contextual effect” caused by uneven built environments and public service provision. “Time poverty” is now a general form of social constraint that cuts across different geographical boundaries, while the transcendent power of cultural capital proves that Bourdieu’s theory is applicable to health behavior studies, revealing the cultural reproduction mechanism behind health inequality. Discovery of the ‘gym paradox’ challenges the simplistic assumption that commercialised fitness is equivalent to modern sporting participation, suggesting instead that we need to reconsider what place there might be for localised forms of exercise which are oriented around lifestyles as part of a national programme on fitness.

## 1 Introduction

Health is an essential requirement for promoting the all-round development of individuals and a fundamental condition for economic and social development. In 2016, the “Healthy China 2030” Outline, issued by the Central Committee of the Communist Party of China and the State Council, elevated national fitness to a core component of the national strategy. The report of the 19th National Congress of the Communist Party of China explicitly proposed “advancing the construction of Healthy China. Prioritize the protection of people’s health in strategic development and improve policies for promoting public health. Further advance the Healthy China initiative and the patriotic health campaign, fostering a culture of civilized and healthy living.” As a cost-effective and highly beneficial preventive health measure, physical activity is recognized as a key strategy to tackle prevalent social issues, including the high prevalence of chronic diseases, the rapid aging of the population, and escalating medical costs. It is supported by evidence that regular exercise can significantly reduce the risk of chronic diseases such as cardiovascular conditions, diabetes, and osteoporosis, and contribute to overall health improvement.

Under the macroscopic picture of the vigorous development of the national fitness cause, the micro-level practice of residents ‘participation in physical activity exhibits profound social stratification characteristics. Although the proportion of Chinese residents who exercise regularly has steadily increased, a huge gap still exists (Liu, 2023) between urban and rural areas, regions, and social classes. The participation rate of urban residents in physical activity is significantly higher than that (Warburton, 2006) of rural residents, and the participation rate of high-income, high-education groups far exceeds that of low-income, low-education groups. This inequality in sports participation is not only a difference in lifestyle but also a concrete manifestation of unequal health opportunities. Regular physical activity can significantly reduce the risk of various chronic diseases and improve life quality (Douglass et al., 2012). Research indicates that social stratification significantly influences residents’ engagement in physical activity, leading to disparities in health outcomes and further entrenching social inequality. On the other hand, contemporary China (Li and Jiang, 2020) is undergoing unprecedented structural changes, which have profoundly reshaped the constraints and opportunities for residents ‘physical activity. First, intense spatial restructuring (Cockerham, 2005). Rapid urbanization has changed the living environment of hundreds of millions of people, but it has also led to a severe imbalance in the spatial distribution of public sports facilities. “Flat type” expansion of new urban areas and the “gentrification” of old urban areas may both alter the accessibility of residents to sports spaces. Second, profound temporal changes. The intensification of market competition and the rise of platform economies have increasingly blurred the boundaries between work and life. The “996” work system and “digital Taylorism” jointly create a widespread phenomenon of “time poverty,” severely squeezing individuals’ leisure and exercise time. Finally, there is the persistent capital divide. While the expansion of higher education has increased the overall cultural capital of the nation, it has also exacerbated the differences in health concepts, physical cognition and lifestyle between different educational groups.

In the transitional period of China, studying the spatial positioning and temporal resource differences in the social stratification mechanism of residents’ physical activity participation is key to revealing the fundamental fairness issue of health inequality among residents, and also provides theoretical support for promoting the precision and fairness of national fitness policies.

## 2 Literature review and research gaps

At present, the research on the influencing factors of physical activity at home and abroad has accumulated fruitful results, which can be roughly divided into three theoretical perspectives: the spatial perspective of built environment and geography, the temporal perspective of sociology and economics, and the capital perspective of social stratification theory:

First, from the spatial perspective of the built environment and geography. This perspective emphasizes the shaping role of the physical space environment on physical activity. Numerous studies have confirmed that elements of the built environment, such (Sallis et al., 2006) as the accessibility of sports facilities, the pedestrian-friendliness of communities, and the quality and safety of park green spaces, are all important factors influencing residents’ participation in sports. In China, scholars have found that urban public sports facilities exhibit significant spatial mismatch issues, such as marked differences in accessibility among different types of public sports facilities. While individual-level factors like gender are known determinants of physical activity (Mclaughlin et al., 2025), our focus is on these broader structural factors. The accessibility of public sports facilities at the block level shows a spatial characteristic of “high in the middle, low on the periphery,” while that at the community level is characterized by scattered distribution. However, most of these studies focus on objective measurements of the built environment, with few systematic explorations from the theoretical perspective of “spatial justice” on how unequal spatial resource allocation, as a structural force, reproduces social inequality in health behaviors.

Second, from the temporal perspective of sociology and economics. This perspective focuses on time as a scarce resource and how its allocation constrains individual behavioral choices. Time-use studies generally find that working hours (Ma and Rhodes, 2011) represent the primary “opportunity cost” of physical activity, with prolonged work significantly encroaching on exercise time. In recent years, with the development of time sociology, researchers have begun (Ruggles and Williams, 1989) to examine the profound impact of “time poverty” on health behaviors. Time poverty not only refers to the absolute lack of free time but also includes low autonomy in time management, fragmented time rhythms, and intense feelings of time pressure. In the context of China, factors such as the “996” work culture, commuting congestion, and family caregiving burdens collectively exacerbate residents’ time pressure. However, few studies have adopted “time equity” as a core analytical dimension to systematically investigate how time deprivation becomes a universal mechanism that transcends different social groups and shapes health inequalities.

Third, the capital perspective based on social stratification theory. This perspective mainly draws on Bourdieu’s capital theory to explore the role of economic capital and cultural capital in shaping healthy lifestyles. Traditional studies often use income (Lu and Miao, 2024) and education as comprehensive indicators of socioeconomic status, finding a strong positive correlation between economic status and sports participation. However, the (Ren and Liu, 2022) essence of Bourdieu’s theory lies in distinguishing the unique mechanisms of different forms of capital. Cultural capital, especially the body concepts and health literacy internalized through education, is considered key to shaping healthy lifestyle choices. Some studies attempt to differentiate the relative importance of economic capital and cultural capital, but the conclusions are not yet unified. In China, the rapid expansion of education has made the connotation and mechanism of cultural capital more complex. Clarifying the independent effects and relative strengths of cultural capital, particularly educational and digital cultural capital, and economic capital in influencing sports participation holds significant theoretical and practical implications.

In summary, existing research provides an important foundation for understanding the social determinants of physical activity, but there are still the following research gaps: First, the fragmentation of theoretical perspectives. The three dimensions of space, time, and capital are often studied in isolation, lacking an integrated analytical framework to examine how they interweave and jointly influence individual physical practice. Social reality is multidimensional, and residents ‘exercise decisions are shaped by the interplay of specific spatial contexts, temporal constraints, and capital endowments. Secondly, there is a lag in research contexts. Most studies rely on outdated data and do not sufficiently account for the significant transformations in China’s spatial, temporal, and capital structures in recent years. Finally, there is insufficient exploration of underlying mechanisms. Current research predominantly focuses on describing correlations, without systematic empirical validation of deeper causal relationships. Mechanisms such as the “component-background” decomposition of spatial effects, the universality of temporal constraints, and the transcendent status of cultural capital.

To address these limitations, this study aims to develop a three-dimensional analytical framework integrating “spatial justice, temporal equity, and cultural capital.” Using the latest CGSS2023 national representative data, we systematically investigate the following core questions: (1) How does spatial location shape disparities in physical activity participation? To what extent are these differences attributable to individual variations (component effects), and to what extent to geographical contexts (background effects)? (2) What is the impact of temporal constraints on physical activity? Is this influence universal across spatial boundaries? (3) Which plays a more significant role in promoting physical activity participation: cultural capital or economic capital? (4) What relationship exists between modern fitness methods and residents’ actual exercise frequency? By answering these questions, this study seeks to provide new theoretical perspectives and empirical evidence to deepen sociological understanding of health inequities.

## 3 Theoretical framework and research hypotheses

### 3.1 Spatial justice: the geographical structure of access to sport

The theory of spatial justice originates from Lefebvre’s (1991) concept of spatial production and Harvey’s (1973) critique of urban resource inequality. It posits that space is not a passive vessel for social activities but a social construct imbued with power dynamics. The organization of space and the geographical distribution of resources profoundly reflect and perpetuate social inequality. Montessori (2011) systematically articulated the concept of spatial justice, defining it as the pursuit of equitable distribution of public resources and life opportunities across spatial dimensions.

Applying the theory of spatial justice to sports participation research requires attention to the spatial distribution of sports resources, a key public good. In China, the distribution of sports facilities exhibits significant multiple spatial inequalities: (1) Urban–rural disparity: Urban areas possess large sports venues, commercial gyms, and high-quality parks and green spaces, while vast rural areas have long suffered from insufficient numbers, limited variety, and poor maintenance of sports facilities (Wang and Qiao, 2013); (2) Regional disparity: The per capita area of sports facilities in the developed eastern coastal regions of China is much higher than in the (Liu and Zhao, 2015) central and western regions, showing a clear gradient decrease from east to west; (3) Internal urban disparity: Within large cities, high-quality sports resources are often concentrated in the city center or upscale communities, while residents in urban–rural fringe areas, old residential neighborhoods, and affordable housing zones face the dilemma of “sports deserts”.

This disparity in spatial resource allocation creates a “geographical opportunity structure” for sports participation. According to the latest data from the National Sports General Administration, as of December 31, 2024, China has a total of 484.17 million sports facilities, with an average sports facility area of 3.0 square meters per capita. This substantial number of sports facilities across the country provides residents with a wide range of exercise options, facilitating the translation of exercise intentions into real-world actions. Conversely, residents in areas with insufficient sports amenities encounter greater challenges in maintaining an active lifestyle. It can be hypothesized that as people migrate from rural areas to urban centers, their participation in sports activities is expected to demonstrate systematic growth.

The mechanisms by which residential location affects outcomes are multifaceted, potentially encompassing a variety of social factors. Notably, different socioeconomic groups exhibit a tendency to cluster in distinct regions. Urban centers draw a higher concentration of highly educated and affluent populations, with contextual effects also potentially contributing. In essence, factors such as the built environment, public services, and social atmosphere in different regions exert independent influences impacts on all residents, regardless of individual characteristics. Clarifying these regional disparities will facilitate the development of spatial intervention policies. Given these considerations, this paper proposes the following hypothesis:

*Hypothesis 1a* (spatial gradient): Different types of residences, such as urban family structures and community types, have been shown to significantly influence the frequency of physical activity participation. From rural to town, urban fringe and urban center, the frequency of physical activity participation gradually increases.

*Hypothesis 1b* (background effect): After controlling for socioeconomic characteristics, the type of residence significantly influences physical activity participation, indicating that spatial factors have independent background effects on sports engagement.

### 3.2 Time equity: time deprivation in an accelerating society

Time sociology views time as a socially constructed scarce resource, where its allocation and utilization reflect social power dynamics. The “social acceleration theory” proposed by German sociologist Rosa (2013) has significantly contributed to understanding contemporary society’s temporal dilemmas. She argues that the tripartite acceleration mechanism—driven by technological advancement, social transformation, and the accelerated pace of life—has plunged modern individuals into a paradoxical experience of (Rosa, 2013) “increasingly insufficient time.” In this hyper-accelerated social environment, time has become a new stratification metric. The ability to possess and autonomously control “time sovereignty” has emerged as a key indicator of social status.

“Time poverty” is a central concept in time inequality research, referring to the state where individuals face extreme scarcity of free time due to excessive time consumed by essential activities like work, household chores, and commuting. The impact of time poverty on health behaviors manifests in three dimensions: (1) Direct deprivation: physical activity requires dedicated continuous time, which directly limits access to exercise opportunities; (2) Mental exhaustion mechanism: High-intensity work drains both physical and mental energy, leaving individuals with “willingness but inability” to choose passive rest during leisure (Vickery, 1977) time; (3) Rhythm disruption effect: Irregular work schedules (such as shift work and hidden overtime under flexible employment systems) disrupt established routines, making it difficult to develop exercise habits.

China’s “time poverty” problem is particularly prominent. The overtime culture represented by “996,” workplace competition, the widespread phenomenon of workers being forced to extend their working hours due to the “high effort-low reward (Sun, 2025)” cost of living, and the blurred boundaries between work and life caused by mobile internet technology have formed a common deprivation scenario. Time poverty may be a more common life barrier than economic poverty. Therefore, the extension of working hours is one of the factors limiting sports participation.

Another critical consideration is whether the effects of time constraints differ across spatial contexts. One plausible scenario is that urban areas with better amenities (24-h gyms) and convenient services (takeout delivery) can offset the impact of time pressure, resulting in less negative effects of working hours on labor income in cities compared to rural areas. Alternatively, time constraints might represent a more fundamental structural force whose adverse effects are universal and cannot be mitigated by spatial resources. This leads us to propose the following hypothesis:

*Hypothesis 2a* (Time compression): Working hours have a significant negative effect on exercise participation frequency, with longer working hours correlating with lower exercise frequency.

There are two kinds of mechanisms that work hours have a negative impact on physical activity, including direct and indirect effects. The direct effect is mainly reflected in the effect of time being squeezed.

### 3.3 Cultural capital: class habits of healthy lifestyles

Bourdieu’s theory of capital provides an excellent framework for understanding class disparities in healthy lifestyles. He argues that social stratification arises not only from economic capital (money and assets), but also from the combined effects of cultural capital (knowledge, taste, education) and social capital (Kayantaş et al., 2022). Cultural capital manifests in three forms: internalized, objectified, and institutionalized.

In the realm of health, the mechanisms of cultural capital exert profound influence. Building upon Bourdieu’s theoretical framework, Cockerham (2005) developed the “Healthy Lifestyle Theory,” which posits that health behaviors such as diet, exercise, and smoking cessation are not merely individual choices, but rather systematic survival practices deeply intertwined with social class positioning. High-culture-capital groups—those who attain social status through advanced education—naturally develop the belief that “the body requires investment and meticulous care” during their formative years. These individuals possess greater (Cockerham, 2005) access to health information, stronger future-oriented thinking, and the ability to translate abstract health concepts into concrete daily practices. The internalized “health habits” embedded in their bodies lead them to consciously or unconsciously favor healthier choices in life decisions.

Conversely, economic capital (income) may exert a more direct and utilitarian influence on sports participation, primarily manifested through financial means such as gym memberships and sports equipment. However, wealth does not equate to health. Cultural capital often proves more significant and enduring than financial means. A highly educated intellectual with a modest income is more likely to maintain regular exercise routines than a newly wealthy tycoon lacking health consciousness. Therefore, understanding the relative weight of cultural and economic capital is crucial for comprehending health stratification. Based on this premise, the paper proposes the following hypotheses:

*Hypothesis 3a* (cultural capital effect): Education level has a significant positive effect on physical activity participation frequency, with higher education level leading to higher physical activity participation frequency.

*Hypothesis 3b* (relative importance of capital): When both economic income and education level are controlled in the model, the influence of education level on physical activity participation is independent of and stronger than economic income.

## 4 Study design

### 4.1 Data source

The data source of this study is the 2023 CGSS (China General Social Survey, CGSS2023). The Chinese General Social Survey (CGSS), initiated in 2003 by the China Survey and Data Center of Renmin University of China, is China’s pioneering national, comprehensive, and continuous large-scale social survey. The primary purpose of this survey was to systematically and comprehensively obtain various data on Chinese people and all aspects of Chinese society, providing high-quality data support for social science research and government decision-making in China, which has gained high reputation in the academic community both domestically and internationally.

The CGSS2023 survey utilized a multi-stage stratified probability sampling (PPS) approach, ensuring that the sample accurately reflects the national adult population aged 18 and above. The survey encompassed all 31 provinces, autonomous regions, and municipalities directly under the central government. The study focused on adults aged 18–70. After removing missing values from dependent and core independent variables, the final valid sample size for analysis was 2,221. In the complete model incorporating all control variables, the sample size was adjusted to 2,129 due to incomplete data on some control variables.

### 4.2 Variable measurement

Dependent variable: Exercise participation frequency. The CGSS2023 questionnaire measures this through the question: “How often have you engaged in physical activity in the past year?” Individuals have engaged in a diverse range of physical activities, such as running, ball games, fitness, swimming, dancing, and other forms of exercise.” Options range from “1 = Never” to “5 = Very frequently (almost daily)”, with responses coded as “2 = Rarely (a few times a year),” “3 = Sometimes (a few times a month)”, and “4 = Often (a few times a week).” This is a typical ordinal categorical variable, where higher scores indicate more frequent exercise participation.

Core independent variable:

1. Residence type (space): the content coding of the answer to the question “Where do you currently live?” is divided into four categories: “1 = Rural,” “2 = Town,” “3=Urban fringe or urban-rural transition area”, and “4=Urban center area”. This approach better reflects the complexity and gradient of the China urban–rural continuum than the traditional urban–rural dichotomy.
2. Working hours (time): measure respondents’ “average weekly working hours”. This is a continuous variable, where higher values indicate longer working hours and stronger time constraints.
3. Years of education (educational qualification): the maximum academic qualifications are converted as follows: no schooling = 0 years, primary school = 6 years, junior high school = 9 years, high school/technical school = 12 years, college = 15 years, undergraduate or above = 16 years.
4. Economic income (economic capital): the natural logarithm of an individual’s annual total income. Given the severe right-skewed distribution of personal annual income data, taking the logarithmic transformation is instrumental in approximating a normal distribution, particularly for positively skewed data. This process reflects the diminishing marginal returns phenomenon, where higher values (such as incomes) yield progressively smaller incremental benefits. In logarithmic calculations, a constant 1 is often added to the annual income before the computation to ensure accurate results.

Controlled variable: To more accurately estimate the net effect of core independent variables, this study controlled for a series of individual characteristics that may affect physical activity:

Demographic variables: gender (male = 1, female = 0), Age (continuous variable).
Methods of sports participation: gym usage. The measurement was conducted through the question “Do I often go to a specialized sports venue or gym to exercise?” with options ranging from “1 = very much” to “4=very much not”. For ease of interpretation, we reverse-coded the values, where higher numbers indicate more frequent gym visits.
Mental and physical state variables: self-rated health status (1 = very unhealthy to 5 = very healthy), life happiness (1 = very unhappy to 5 = very happy). These variables are the results of physical activity, but may also affect the motivation and ability to do physical activity in reverse, so they can be included in the model to control for endogeneity.

### 4.3 Analytical strategy

Given the ordinal nature of the dependent variable (exercise frequency from “Never” to “Almost daily”), an ordered logistic regression model was employed for the analysis. We constructed a series of nested models to examine the distinct and combined associations of our core independent variables with physical activity participation. To ensure the model’s validity, we conducted a Brant test on the full model (Model 8) to check the proportional odds (parallel lines) assumption. The test results confirmed that the assumption was met (*p* > 0.05), validating the use of this model.

## 5 Empirical results analysis

### 5.1 Descriptive statistics

Prior to initiating model analysis, we conducted a thorough descriptive statistical analysis on the sample data (refer to Table 1). The mean exercise frequency was determined to be 2.535, indicating a moderate level of activity. “Rarely” and “occasionally,” with a standard deviation of 1.526, indicating significant variations in residents ‘exercise habits. The average residence type was 2.172, while weekly working hours reached 49.47 h—far exceeding the 40-h weekly limit stipulated by China’s Labor Law—highlighting severe overwork. The sample’s average education duration was 9.78 years, roughly equivalent to completing junior high school.

**Table 1:** Descriptive statistics of variables.

Variable	Sample capacity	Mean	Standard deviation	Remarks
Frequency of physical activity participation among residents	2,129	2.535	1.526	1 = Never, 5 = Almost every day
Type of residence	2,129	2.172	1.257	1 = rural, 4 = urban center
Operating time	2,129	49.474	22.193	Average weekly working hours
Years of education	2,129	9.783	4.344	0–16 years
Economic income (logarithm)	2,129	10.259	1.247	Logarithm of personal annual income
Gender (male = 1)	2,129	0.510	0.500	
Age	2,129	48.381	13.066	One full year of life
Use gym (reverse)	2,129	1.543	0.793	1 = Not at all, 4 = Very much
Health condition	2,129	3.652	1.019	1 = Very unhealthy, 5 = Very healthy
Happiness	2,129	3.916	0.794	1 = Very unhappy, 5 = Very happy

### 5.2 Results of the ordered logistic regression model

Table 2 presents the results of eight nested ordered logistic regression models, which were utilized to test the research hypotheses by examining the impact of various factors on sports participation among Chinese residents.

**Table 2:** Results of the ordered logistic regression model for residents’ exercise participation frequency.

Variable	Model 1	Model 2	Model 3	Model 4	Model 5	Model 6	Model 7	Model 8
Type of residence (reference: rural)	−	−	−	−	−	−	−	−
Town	0.981*** (0.126)	0.772*** (0.134)	0.559*** (0.136)	0.588*** (0.137)	0.594*** (0.137)	0.506*** (0.138)	0.500*** (0.138)	0.502*** (0.139)
Urban fringe	1.034*** (0.109)	0.798*** (0.119)	0.591*** (0.122)	0.631*** (0.122)	0.634*** (0.122)	0.511*** (0.123)	0.494*** (0.123)	0.502*** (0.124)
Downtown	1.344*** (0.098)	1.097*** (0.110)	0.815*** (0.114)	0.881*** (0.116)	0.880*** (0.116)	0.702*** (0.118)	0.706*** (0.118)	0.729*** (0.119)
Operate time	−0.006*** (0.002)	-0.006*** (0.002)	−0.005** (0.002)	−0.004** (0.002)	−0.004** (0.002)	−0.003 (0.002)	−0.003* (0.002)	−0.003* (0.002)
Economic income (logarithm)		0.206*** (0.038)	0.063 (0.041)	0.047 (0.041)	0.066 (0.042)	0.036 (0.043)	0.018 (0.043)	0.014 (0.043)
Years of education			0.106*** (0.012)	0.107*** (0.012)	0.115*** (0.013)	0.109*** (0.013)	0.109*** (0.013)	0.106*** (0.013)
Gender (male = 1)				0.267*** (0.082)	0.235*** (0.084)	0.167** (0.084)	0.168** (0.084)	0.177** (0.084)
Age					0.007* (0.004)	0.011*** (0.004)	0.013*** (0.004)	0.012*** (0.004)
Available at the gym (opposite direction)						−0.504*** (0.054)	−0.490*** (0.054)	−0.481*** (0.054)
Health condition							0.160*** (0.044)	0.139*** (0.045)
Happiness								0.117** (0.057)
Observations	2,129	2,129	2,129	2,129	2,129	2,129	2,129	2,129
Pseudo R^2^	0.035	0.040	0.053	0.054	0.055	0.069	0.071	0.071

The values in parentheses represent the robust standard error; *** *p* < 0.01, ** *p* < 0.05, * *p* < 0.1.

#### 5.2.1 Spatial gradient effect and background effect

Model 1 serves as the baseline model, incorporating only residence type and working hours. Recent data and case studies clearly illustrate the widespread spatial gradient effect of physical activity participation, with significant increases in participation rates across all age groups, particularly among the youth and elderly. Using rural residents as the reference group, residents in towns, urban fringe areas, and city centers exhibited significantly higher exercise frequencies. The regression coefficients were 0.981 for town residents, 1.034 for urban fringe areas, and 1.344 for city centers, all statistically significant at *p* < 0.001. By transforming these coefficients into odds ratios (OR = e^*β*), we gain a more intuitive understanding: town residents had a 2.67-fold increased likelihood compared to rural residents to participate in high-frequency activities. Exercise (e^0.981), urban fringe residents 2.81 times more likely, and city center residents 3.84 times more likely. This distinct, progressively increasing gradient difference strongly supports Hypothesis 1a.

To test Hypothesis 1b, we investigated the statistical significance of residential location coefficients after sequentially adding individual-level control variables to our regression model. In the final full model (Model 8), we observed that although the coefficient of urban centers decreased from 1.344 to 0.729, these values remained highly significant at the *p* < 0.001 level. Studies have shown that, even after accounting for personal factors such as income, education, gender, and age, the location where an individual resides can independently and significantly influence their level of physical activity. Specifically, the attenuation of the urban center coefficient from 1.344 to 0.729 after including individual-level controls allows for a heuristic approximation of these effects. This heuristic calculation, while not a formal decomposition, suggests that approximately 45.7% of the initial gap is associated with differences in population composition. The remaining 54.3% can be interpreted as an approximate measure of the independent contextual “background effect.” This suggests that spatial factors are associated with physical activity as structural forces independent of individual attributes, thereby supporting Hypothesis 1b.

#### 5.2.2 The universal effect of time constraints

Working hours were found to have a statistically significant negative association across most models, validating Hypothesis 2a. The benchmark model 1 coefficient of−0.006 (*p* < 0.001) indicates that as the average weekly working hours in China have increased, with the national average reaching 49 h in 2023, each additional hour of work per week is associated with a 0.6% reduction in the odds of being in a higher exercise frequency category. Although this reduction may seem minor on an individual level, the aggregate impact over time can be significant. For example, a “996” worker (a 60-h workweek schedule) has approximately 11.3% lower probability of participating in high-frequency exercise compared to a standard 40-h worker, calculated through the formula 1-e^(−0.006*20).

Notably, when health status and happiness were incorporated into Models 7 and 8, the absolute values of the working hours coefficient decreased, with its significance also showing a moderate decline. In Model 8, the coefficient was-0.003 (*p* < 0.1), indicating that the negative impact of working hours on physical activity stems partly from its effect on individuals’ physical and mental well-being. When people feel physically exhausted and emotionally distressed due to excessive workloads, they naturally lack both motivation and energy to exercise. This finding partially validates the mediating role proposed in Hypothesis 2b.

#### 5.2.3 The effect of cultural capital over economic capital

The results from Model 2 and Model 3 provide strong evidence to validate Hypotheses 3a and 3b. When Model 2 incorporates economic income (log-transformed) alone, its coefficient of 0.206 (*p* < 0.001) is significantly positive, indicating that higher income correlates with more frequent exercise—a finding consistent with common understanding. However, dramatic changes emerge when Model 3 adds years of education: the coefficient for economic income plummets to 0.063 and loses statistical significance (*p* > 0.1), while the coefficient for years of education surges to 0.106 (*p* < 0.001), demonstrating a robust and consistent positive effect that remains highly stable across all subsequent models.

Model 8 reveals that the advantage of participating in higher-frequency exercise increases by approximately 11.2% (e^0.106) for each additional year of education. Specifically, individuals with a bachelor’s degree (16 years of education) are about 119% more likely to engage in high-frequency exercise than those with junior high school education (9 years of education), after controlling for all other variables (e^(0.106*7)−1). This represents more than double the odds of the latter group, clearly suggesting that education-centered cultural capital has an association with healthy lifestyles that is independent of and stronger than that of economic capital. This strongly confirms Hypotheses 3a and 3b.

#### 5.2.4 “The gym paradox” and other findings

Model 6 introduced the “gym usage” variable, yielding an unexpected yet enlightening result: the coefficient of “gym usage” was-0.504 (*p* < 0.001), indicating a significant negative correlation. This means that individuals who claim to “regularly exercise at the gym” actually have much lower overall physical activity levels. We refer to this phenomenon as the “gym paradox,” which Contrary to the traditional notion, modern facilities like gyms are increasingly associated with high levels of physical participation. Possible explanations for this will be elaborated in the discussion section.

The results of other control variables align with expectations. Research indicates that men tend to exercise more frequently than women, and there is a weak yet significant positive correlation between age and exercise frequency, with older individuals showing a higher participation rate in physical activities. This may be attributed to the gradual increase in health awareness with age or the expanded leisure after retirement, both self-reported physical health and happiness levels exhibit positive correlations with exercise frequency. Individuals with good physical and mental health are more likely to choose physical activity.

### 5.3 Findings and discussion

This study examines the issue from the perspectives of spatial justice and temporal equity, analyzing the social stratification logic underlying Chinese residents’ participation in physical activity. It not only confirms the explanatory power of classical sociological theories but also provides a new perspective for interpreting health inequality in China during its transitional period.

First, the imprint of space: the geographical location as an opportunity structure. Research confirms a clear spatial gradient in sports participation, with residents ‘exercise opportunities and engagement levels systematically improving from rural to urban centers. More importantly, our model decomposition reveals that over half of the spatial effects are “background effects” independent of individual characteristics, strongly indicating the significance of “local context.” Urban centers promote sports participation not merely by attracting elite populations, but by providing a supportive “health ecosystem” featuring dense sports facilities, safe public spaces, convenient transportation networks, and a culture that views fitness as fashionable and normalized. Conversely, rural areas face not only “hard constraints” in facilities but also a lack of “soft environments” encouraging recreational sports. This finding reveals that spatial injustice in resource allocation directly translates into health inequality through shaping residents’ daily opportunities, thereby reinforcing health disparities between social classes. It reminds us that national fitness policies must prioritize spatial sensitivity, focusing on eliminating the “sports divide” and allocating resources to rural and urban fringe areas.

Second, the shackles of time: universal structural deprivation. A significant finding from recent research indicates that long working hours universally impede physical activity, a trend that persists even in urban areas with improved spatial resources. This “spatially homogeneous” phenomenon indicates that time constraints have structural and fundamental characteristics. In contemporary China, the efficiency-centered social acceleration logic has permeated every aspect of social life, reshaping the cultural structure and lifestyle, and influencing the meaning of life for young people, as well as the production and dissemination of media content. Being thoroughly commodified and workers ‘time sovereignty severely violated. Whether it is the “996” and “KPI” faced by urban white-collar workers or the piece-rate wages and unstable labor relations encountered by rural workers, the essence is that individuals’ time is controlled by the production system. When survival pressures force individuals to devote large amounts of time to work, physical activity becomes a luxury. Therefore, promoting physical activity participation cannot remain at the level of “advocacy” and requires reforms in labor systems and time policies, safeguarding workers’ rights to rest, opposing the “overwork culture” stemming from unlimited overtime, and promoting more flexible work arrangements. This constitutes the fundamental approach to providing basic conditions for national fitness.

Thirdly, the logic of capital: How can culture transcend economic constraints? One of the most compelling findings of this study demonstrates that cultural capital exhibits a stronger predictive power in influencing students’ academic outcomes. Power than economic capital in explaining sports participation, profoundly revealing the class reproduction of healthy lifestyles. Education provides not only survival skills and higher incomes but also “habitus” (habitual patterns) for physical health and living. Highly educated individuals tend to view their bodies as a “self-project” requiring long-term planning, scientific management, and continuous investment. With cognitive abilities and self-discipline, they transform abstract health knowledge into daily practices. Income growth merely removes economic barriers without necessarily forming healthy “habitus.” This discovery offers strong empirical support for Bourdieu’s theory while challenging health promotion practices: Relying solely on income increases is insufficient. We must enhance public health literacy and cultural capital, requiring health education to be integrated throughout the national education system to cultivate scientific health concepts and positive lifestyles from childhood.

Fourth, a reflection on sports modernity: Unpacking the “apparent gym paradox”. The finding that more frequent gym use is associated with lower overall physical activity frequency presents an empirical anomaly, which we term the “apparent gym paradox.” This counterintuitive result does not necessarily imply that gyms hinder exercise; rather, it prompts a more nuanced reflection on the relationship between commercialized fitness and overall activity levels. Several alternative explanations, which our cross-sectional data cannot definitively disentangle, must be considered.

First, selection bias may be at play: individuals who perceive themselves as having low exercise motivation or self-discipline might be more inclined to purchase gym memberships, hoping the financial investment will compel them to be active. However, these same individuals may be less likely to maintain regular exercise habits over the long term. Second, reverse causality could be a factor: people who are already struggling to find time or opportunities for other forms of exercise may turn to gyms as a last resort, meaning their low overall frequency precedes their gym attendance. Third, a substitution effect could exist, where the high investment of time and money in gym visits might displace other more accessible, lifestyle-integrated activities like walking or community sports. Finally, a cultural mismatch may contribute, where the goal-oriented, body-centric culture of many modern gyms may not align with the health-preservation and social-interaction motivations prevalent in traditional Chinese exercise culture.

This finding cautions against a simplistic equation of sports modernization with commercial gym proliferation. It suggests that in promoting national fitness, it is crucial to support a diverse ecosystem of physical activities, including accessible, community-based, and culturally resonant forms like square dancing, Tai Chi, and local sports, which may form the bedrock of sustainable physical activity for a broader population.

## 6 Conclusion and policy implications

### 6.1 Main conclusions

This study, based on the CGSS2023 national representative data, constructs and validates an analytical framework integrating spatial, temporal, and capital dimensions, yielding four key conclusions: Firstly, a significant spatial stratification in physical activity participation is observed, with a clear gradient effect from rural to urban areas, as evidenced by the participation rates among urban and rural residents in China. This effect operates independently of individual characteristics, revealing strong “contextual effects” that highlight the value of spatial justice in health. Second, working hours serve as a universal structural constraint affecting physical activity participation, with their negative impacts extending across urban and rural spaces. “Time equity” is therefore essential for achieving universal health. Third, cultural capital proves more crucial than economic capital, as education shapes health “habitus” that profoundly influences physical practices, explaining the cultural reproduction of health inequalities. Fourth, the emergence of the “gym paradox” challenges the linear narrative of sports modernization, sounding an alarm for policymakers to prioritize localized, lifestyle-oriented, and community-based exercise models.

### 6.2 Policy implications

Based on the above conclusions, this study puts forward the following three policy implications: Fourth, a reflection on sports modernity: Unpacking the “apparent gym paradox”.

The finding that more frequent gym use is associated with lower overall physical activity frequency presents an empirical anomaly, which we term the “apparent gym paradox.” This counterintuitive result does not necessarily imply that gyms hinder exercise; rather, it prompts a more nuanced reflection on the relationship between commercialized fitness and overall activity levels. Several alternative explanations, which our cross-sectional data cannot definitively disentangle, must be considered.

First, selection bias may be at play: individuals who perceive themselves as having low exercise motivation or self-discipline might be more inclined to purchase gym memberships, hoping the financial investment will compel them to be active. However, these same individuals may be less likely to maintain regular exercise habits over the long term. Second, reverse causality could be a factor: people who are already struggling to find time or opportunities for other forms of exercise may turn to gyms as a last resort, meaning their low overall frequency precedes their gym attendance. Third, a substitution effect could exist, where the high investment of time and money in gym visits might displace other more accessible, lifestyle-integrated activities like walking or community sports. Finally, a cultural mismatch may contribute, where the goal-oriented, body-centric culture of many modern gyms may not align with the health-preservation and social-interaction motivations prevalent in traditional Chinese exercise culture.

This finding cautions against a simplistic equation of sports modernization with commercial gym proliferation. It suggests that in promoting national fitness, it is crucial to support a diverse ecosystem of physical activities, including accessible, community-based, and culturally resonant forms like square dancing, Tai Chi, and local sports, which may form the bedrock of sustainable physical activity for a broader population. The focus of creating “15-minute fitness circles” should be placed in resource-deficient areas. Regarding facility types, we should adopt location-specific solutions by building community-proximate, cost-effective, and practical facilities like walking paths, sports courts, and multi-purpose venues, rather than blindly pursuing “high-end” sports stadiums.

Second, safeguarding workers “‘right to exercise time’ by incorporating citizens” fitness time into labor supervision and corporate social responsibility evaluations. On one hand, strictly enforce the Labor Law of the People’s Republic of China to effectively regulate excessive overtime practices like the “996” work schedule. Promote and encourage enterprises and institutions to implement health management systems, establish “work-break exercises,” offer fitness subsidies and implement flexible working hours. Acknowledge employee health as a vital organizational asset.

Third, our findings suggest the importance of deepening cultural development centered on enhancing health literacy by fully integrating health education into both basic and higher education systems. This initiative is designed to foster citizens’ health awareness and self-management skills, while also promoting diverse sports cultures. This could involve not only advancing modern sports but also supporting the preservation of traditional disciplines. By organizing community sports festivals and family sports events, we can create a lively atmosphere that promotes nationwide fitness participation.

### 6.3 Research limitations and prospects

This study also has certain limitations. Firstly, the data utilized in this study are cross-sectional, which precludes strict causal inference. In the future, panel data could be employed to investigate the influence process of various factors more deeply. In terms of variable measurement, the frequency of physical activity relies on respondents’ self-reporting, which may lead to social desirability bias. In the future, wearable devices, with their objective data capabilities, can be integrated to validate this approach. This study focuses on macro-structural factors, and the exploration of micro-mechanisms such as individual motivation, preferences, and social networks is not sufficiently addressed. This can be supplemented in the future through qualitative interviews and other methods. Despite these limitations, this study still provides valuable references for understanding the complex mechanisms of health inequality in China through its integrated theoretical perspective and rigorous empirical analysis, and points out feasible directions for building a more equitable and healthier society.

## Data Availability

The original contributions presented in the study are included in the article/supplementary material, further inquiries can be directed to the corresponding author.
